# Burden of suicide presented as one of the leading causes of death: uncover facts or misrepresent statistics?

**DOI:** 10.7189/jogh.09.010401

**Published:** 2019-06

**Authors:** Finn Gjertsen, Silvia Bruzzone, Clare E Griffiths

**Affiliations:** 1 Department of Mental and Somatic Health, Norwegian Institute of Public Health, Oslo, Norway; 2 Directorate for Social Statistics and Population Census, National Institute of Statistics, Rome, Italy; 3 Health Improvement Directorate, Public Health England, London, UK

## Abstract

**Background:**

Suicide is a relatively rare incident. Nevertheless, parts of the literature on intentional self-harm behaviour state that suicide is one of the leading causes of death. We aimed to assess the evidence behind the statement that suicide is a leading cause of death across all ages, with reference to the methods of ranking causes of death.

**Methods:**

Two sets of data were used: For the European Union (EU) we used cause specific mortality statistics from the European Statistical Office (Eurostat) for the data-year 2014, and globally and for the WHO European Region we used data from Global Health Estimates (GHE) 2015. We used different sets of rules to select mutually exclusive leading underlying causes of mortality for Europe (EU28). We also present lists with estimates of leading causes of death globally, and for the WHO European Region based on the GHE 2015.

**Results:**

In 2014, 1.2% of all reported deaths in the Europe Union (EU28) were due to suicide, and 1.4% globally (2015) according to the WHO estimates. In Europe, suicide was ranked as number 11 and 15 in the two different ranking lists we used, and according to GHE-2015, suicide was ranked as the 17^th^ leading cause globally, and number 14 in the WHO European Region. Looking at the differences by sex, suicide for males was ranked as the ninth and the tenth leading cause of death in two ranking lists for the European Union. For females, suicide was number 13 in the first and 23 in the second list, respectively.

**Conclusions:**

Different cause lists and rules for ranking produce different leading causes of mortality. The quality of data can also affect the ranking. Our rankings suggested that suicide was not among the ten leading causes of death in Europe or globally. To ensure that ranking causes of death is not driven by political motives and funding considerations, standard methods and official tabulation lists should be used. The rankings do not necessarily present the causes of mortality of greatest public health importance.

Several recent research studies on suicidal behaviour have reported that suicide is a major or one of the leading causes of mortality at all ages, nationally or worldwide [1-5]. We may find similar descriptions in other type of documents, for example in the first Norwegian suicide prevention plan for the period 1994-1998 [6]. This plan stated that suicide was the second most important (leading) cause of death globally; suicides took more life than war, violence, and human immunodeficiency virus (HIV), and only traffic accidents killed more than suicides [6]. At best, such descriptions and comparisons may increase awareness of the public health significance of suicide and intentional self-harm. Nevertheless, we find reason to ask if such statements present statistical facts, or misrepresent official statistics and exaggerate the relative burden of suicide mortality. The rank order of any disease or injury category in a list depends strongly on the way the causes of death have been aggregated within the list used to select leading causes, ie, the way in which the causes are grouped or split into subcategories will give different leading causes of death [7]. Suicide is a relative rare event, and the category covers all injury mechanisms (eg, poisoning, hanging/suffocation, firearm). This means it is more likely to appear higher up in ranking lists when compared with subcategories of other causes of death, eg, subcategories of accidents or specific diseases. A high score might also be the result of comparing all suicides with some selected causes, and not with all causes in the list. Expending the suicide category, eg, including all events of undetermined intent or most accidental poisoning deaths, motivated by underestimation of suicide, can also give a similar result. Approaches that favour the cause under study may give an invalid and biased list of the leading causes of death.

In recent decades, many countries have implemented suicide prevention activities, involving governmental and non-governmental institutions. In 2013, 28 countries globally reported to the World Health Organization they had a national suicide prevention strategy, and 13 countries had a national strategy under development [8]. It is reasonable to assume that more openness about mental health, the fact that suicide kills relatively many young adults, together with a growing belief that suicide is preventable, supports political decisions on suicide prevention. Such factors seem to be more critical for developing suicide prevention strategies than the frequency of suicide and suicides rates per se; therefore such strategies are introduced in countries where the suicide rates differs significantly. Grouping and ranking causes of death is a method used to illustrate and highlight their relative importance or burden in a population, enhancing arguments for research funding, prevention, and health services and treatment of a specific disease or injury cause [9-12]. In the United States, lists with leading cause-of-death categories has been published since 1952, beginning with 1949 data, on which the first official ranking causes were introduced [13]. Such rankings provide useful information, but have also weaknesses that could disrupt the comparability over time and between populations [9,10]. In England and Wales, statistics on leading causes of death have been published by Office for National Statistics since 2005, after amending the standard WHO list [7] to take account of idiosyncrasies in the recording and coding of cause of death in England and Wales.

The purpose of this article was to assess the evidence behind the statement that suicide is a leading cause of death, with reference to the methods of ranking causes of death used to position a rare incident like suicide as one of the leading causes of death.

## METHODS

### Mortality data from Eurostat

For this study, we accessed cause-of-death statistics from the European Statistical Office (Eurostat) for the data-year 2014. As at the end of March 2017 this included reported data from all the 28 European Union member countries (EU28) [14]. The EU28 includes these member countries as at 1 January 2013: Austria, Belgium, Bulgaria, Croatia, Cyprus, the Czech Republic, Denmark, Estonia, Finland, France, Germany, Greece, Hungary, Ireland, Italy, Latvia, Lithuania, Luxembourg, Malta, the Netherlands, Poland, Portugal, Romania, Slovakia, Slovenia, Spain, Sweden and the United Kingdom.

The mortality statistics published by Eurostat, like other national and international mortality statistics, are based on one single underlying cause of death. If two or more causes are recorded on the form of medical death certificate, one cause should be selected and used for primary tabulation. The cause used to compile official cause-specific mortality statistics should be designated the underlying cause of death, defined as *“(a) the disease or injury which initiated the train of morbid events leading directly to death, or (b) the circumstances of the accident or violence which produced the fatal injury”* [15] (page 31). This definition was introduced in the sixth revision of the International Statistical Classification of Diseases (ICD-6), with the purpose of obtaining essential information for prevention: *“From the standpoint of prevention of death, it is necessary to break the chain of events or to cure at some point. The most effective public health objective is to prevent the precipitating cause from operating”* [15] (page 31).

### Definition of rankable causes

The cause-of-death statistics from Eurostat were grouped according to the extended mortality shortlist of 2012 [14]. This list contains in total 86 cause-categories (including subgroups and groups of residuals) instead that the 65 ones included in the previous version. We used these 86 categories to select two different lists of mutually exclusive causes for this study (**Table 1**). In our first list, we grouped three main categories, which contained subcategories: malignant neoplasms, diseases of the circulatory system, and accidents (unintentional injuries). In the second ranking list, we split these three categories. If a cause category contained subcategories, the most detailed cause-category was selected (except for three main cause-of-death categories in the first list). In the first list, we identified 28 causes, and in the second, 51 causes (**Table 1**). All cause-of-death categories starting with “other”, “symptoms”, “ill-defined”, and “event of undetermined intent” were excluded from the selection of cause categories. The ranking of selected causes was based solely on the number of deaths, and not rates. Crude rates would have given the same result, in contrast to standardized rates because the selection of standard population may impact on the ranking. We mostly followed the procedures used by the US National Center for Health Statistics (NCHS) for ranking causes of death [9,10], with one important exception. We selected causes from the list of 86 causes, the NCHS use a list of 113 causes [9]. We included one residual category, which identifies the percentage of all deaths not covered by the lists of leading causes. In addition to the causes that were ranked, the category “Symptoms, signs and ill-defined conditions”, excluding “Sudden infant death syndrome - SIDS” (ICD-10 codes R00-R99, excl. R95) [16], is presented separately. The proportion of deaths classified to this set of causes, as the underlying cause of death, is one internationally used indicator of the quality of mortality data [12,17,18].

**Table 1:** Lists of rankable and mutually exclusive causes of death categories selected from the European mortality short list used by Eurostat*

Rankable causes		Cause-of-death categories with ICD-10 codes (Eurostat short list)
**List I**	**List II**	
…	…	**All causes of death (A00-R99, V01-Y89)**
…	…	**Certain infectious and parasitic diseases (A00-B99)**
1	1	- Tuberculosis (A15-A19, B90)
2	2	- Viral hepatitis and sequelae of viral hepatitis (B15-B19, B94.2)
3	3	- Human immunodeficiency virus [HIV] disease (B20-B24)
…	…	- Other infectious and parasitic diseases (remainder of A00-B99)
…	…	**Neoplasms (C00-D48)**
4	…	- Malignant neoplasms (C00-C97)
…	4	Malignant neoplasm of lip, oral cavity, pharynx (C00-C14)
…	5	Malignant neoplasm of oesophagus (C15)
…	6	Malignant neoplasm of stomach (C16)
…	7	Malignant neoplasm of colon, rectosigmoid junction, rectum, anus and anal canal (C18-C21)
…	8	Malignant neoplasm of liver and intrahepatic bile ducts (C22)
…	9	Malignant neoplasm of pancreas (C25)
…	10	Malignant neoplasm of larynx (C32)
…	11	Malignant neoplasm of trachea, bronchus and lung (C33, C34)
…	12	Malignant melanoma of skin (C43)
…	13	Malignant neoplasm of breast (C50)
…	14	Malignant neoplasm of cervix uteri (C53)
…	15	Malignant neoplasm of other parts of uterus (C54, C55)
…	16	Malignant neoplasm of ovary (C56)
…	17	Malignant neoplasm of prostate (C61)
…	18	Malignant neoplasm of kidney, except renal pelvis (C64)
…	19	Malignant neoplasm of bladder (C67)
…	20	Malignant neoplasm of brain and central nervous system (C70-C72)
…	21	Malignant neoplasm of thyroid gland (C73)
…	22	Hodgkin disease and lymphomas (C81-C85)
…	…	Other malignant neoplasm of lymphoid, haematopoietic and related tissue (C88, C90, 96)
…	23	Leukaemia (C91-C95)
…	…	Other malignant neoplasms (remainder of C00-C97)
5	24	- Non-malignant neoplasms (benign and uncertain) (D00-D48)
6	25	**Diseases of the blood and blood-forming organs and certain disorders involving the immune mechanism (D50-D89)**
…	…	**Endocrine, nutritional and metabolic diseases (E00-E90)**
7	26	- Diabetes mellitus (E10-E14)
…	…	- Other endocrine, nutritional and metabolic diseases (remainder of E00-E90)
…	…	**Mental and behavioural disorders (F00-F99)**
8	27	- Dementia (F01, F03)
9	28	- Mental and behavioural disorders due to use of alcohol (F10)
10	29	- Drug dependence, toxicomania (F11-F16, F18-F19)
…	…	- Other mental and behavioural disorders (remainder of F00-F99)
…	…	**Diseases of the nervous system and the sense organs (G00-H95)**
11	30	- Parkinson disease (G20)
12	31	- Alzheimer disease (G30)
…	…	- Other diseases of the nervous system and the sense organs (remainder of G00-H95)
13	…	**Diseases of the circulatory system (I00-I99)**
…	…	- Ischaemic heart diseases (I20-I25)
…	32	Acute myocardial infarction including subsequent myocardial infarction (I21, I22)
…	…	Other ischaemic heart diseases (I20, I23-I25)
…	…	- Other heart diseases (I30-I51) (excludes pulmonary diseases, I26-I28)
…	33	- Cerebrovascular diseases (I60-I69)
…	…	- Other diseases of the circulatory system (remainder of I00-I99)
…	…	**Diseases of the respiratory system (J00-J99)**
14	34	- Influenza (including swine flu) (J09-J11)
15	35	- Pneumonia (J12-J18)
16	…	- Chronic lower respiratory diseases (J40-J47)
…	36	Asthma and status asthmaticus (J45, J46)
…	…	Other lower respiratory diseases (J40-J44, J47)
…	…	- Other diseases of the respiratory system (remainder of J00-J99)
…	…	**Diseases of the digestive system (K00-K93)**
17	37	- Ulcer of stomach, duodenum and jejunum (K25-K28)
18	38	- Chronic liver disease (K70, K73, K74)
…	…	- Other diseases of the digestive system (remainder of K00-K93)
19	39	**Diseases of the skin and subcutaneous tissue (L00-L99)**
…	…	**Diseases of the musculoskeletal system and connective tissue (M00-M99)**
20	40	- Rheumatoid arthritis and arthrosis (M05-M06, M15-M19)
…	…	- Other diseases of the musculoskeletal system and connective tissue (remainder of M00-M99)
…	…	**Diseases of the genitourinary system (N00-N99)**
21	41	- Diseases of kidney and ureter (N00-N29)
…	…	- Other diseases of the genitourinary system (remainder of N00-N99)
22	42	**Pregnancy, childbirth and the puerperium (O00-O99)**
23	43	**Certain conditions originating in the perinatal period (P00-P96)**
24	44	**Congenital malformations, deformations and chromosomal abnormalities (Q00-Q99)**
…	…	**Symptoms, signs and abnormal clinical and laboratory findings, not elsewhere classified (R00-R99)**
25	45	- Sudden infant death syndrome (R95)
…	…	- Ill-defined and unknown causes of mortality (R96-R99)
…	…	- Other symptoms, signs and abnormal clinical and laboratory findings (remainder of R00-R99)
…	…	**External causes of morbidity and mortality (V01-Y89)**
26	…	- Accidents (unintentional injuries) (V01-X59, Y85, Y86)
…	46	Transport accidents (V01-V99, Y85)
…	47	Accidental falls (W00-W19)
…	48	Accidental drowning and submersion (W65-W74)
…	49	Accidental poisoning by and exposure to noxious substances (X40-X49)
…	…	Other accidents (W20-W64, W75-X39, X50-X59, Y86)
27	50	- Intentional self-harm (suicide) (X60-X84, Y87.0)
28	51	- Assault (homicide) (X85-Y09, Y87.1)
…	…	- Event of undetermined intent (Y10-Y34, Y87.2)
…	…	- Other external causes of morbidity and mortality (remainder of V01-Y89)

… – Category not applicable

*List I of rankable causes grouped cancers, diseases of circulatory system, and accidents (unintentional injuries). List II split cancers, diseases of circulatory system, and accidents. Only mutually exclusive categories are numbered.

### Global Health Estimates 2015

In addition to data from Eurostat, we have also used summary estimates of leading causes of death for the year 2015, ranked by the World Health Organization (WHO) [19]. The last available estimates contain figures for the four years 2000, 2005, 2010 and 2015 (2000-2015) [19,20]. The data sources behind these estimates are a mix of information from vital registration systems, periodic and non-national-level samples, and disease surveillance systems. Based on all this information, statistical models were used to adjust for completeness, underreporting of specific causes of death, redistribution of unknown information on age, sex and causes of death, and to interpolate and extrapolate where national data was missing for some years. The WHO Global Health Estimates (GHE) 2015 on mortality used a list of, in total, 202 cause-of-death categories [19,20], instead of the 163 covered in the previous GHE for 2014 [21,22]. The estimates of WHO for the years 2000-2015 were based on latest available national information on levels of mortality and cause distributions as at the end of October 2016 together with available information from WHO programs [19]. Because of substantial revisions of the method and data sources behind the global and regional estimates, the WHO estimates for the years 2000-2015 [20] are not directly comparable with previous WHO estimates for 2000-2012 [21] or earlier versions. We present WHO`s global ranking of leading causes of mortality, and for the WHO European Region for the year 2015. The WHO European Region includes 53 countries [20]. In addition to the 28 European Union countries (EU28) listed below, these countries are defined into the region: Albania, Andorra, Armenia, Azerbaijan, Belarus, Bosnia and Herzegovina, Georgia, Iceland, Israel, Kazakhstan, Kyrgyzstan, Monaco, Montenegro, Norway, Republic of Moldova, Russian Federation, San Marino, Serbia, Switzerland, Tajikistan, The former Yugoslav Republic of Macedonia, Turkey, Turkmenistan, Ukraine, and Uzbekistan. It must be added, the WHO regional grouping as of 2015 excluded analyzing information from WHO Member States with a population of less than 90 000 population in 2015 [20]. Regarding the European region, Andorra, Monaco, and San Marino were not included in GHE 2015 [20], but included in the GHE 2014 analysis [21].

### Ethics

This study was solely based on aggregate cause of death statistics for Europe and estimates from the World Health Organization, globally and the WHO European Region. All the data we used was available to the public on the internet, which means that the information was anonymous without any conditions relating to privacy considerations. Therefore, no ethical or governmental permissions were required for this study.

## RESULTS

In 2014, 1.2% of all deaths in the enlarged Europe Union (28 countries) were registered with suicide as the underlying cause of death, and 1.4% of all deaths globally (2015) according to the rough WHO-estimates. Suicide was not ranked among the ten leading causes of death (all ages), neither in Europe (EU-28), nor in the WHO European Region (53 countries), nor globally. In Europe, unintentional injuries (accidents) killed 2.6 times more people than intentional self-harm (suicide) in 2014 (**Table 2**), and the global estimates indicate that accidents killed 4.5 times more people than intentional self-harm (3 527 000 and 788 000 deaths, respectively) [19], in 2015.

**Table 2:** The 10 leading causes of death selected from List I (see Table 1), by sex: 28 European Union member countries (EU-28), 2014

Rank	Grouped: Circulatory diseases; cancers; accidents (list I)	2014	
**Causes of death (ICD-10)**	**Deaths**	**%**
…	**All causes of death (A00-R99, V01-Y89)**	**4 945 366**	**100.0**
1	Diseases of the circulatory system (I00-I99)	1 832 799	37.1
2	Malignant neoplasms (C00-C97)	1 306 561	26.4
3	Chronic lower respiratory diseases (J40-J47)	163 943	3.3
4	Dementia (F01, F03)	158 906	3.2
5	Accidents (V01-X59, Y85, Y86)	152 381	3.1
6	Pneumonia (J12-J18)	118 295	2.4
7	Diabetes mellitus (E10-E14)	108 654	2.2
8	Alzheimer disease (G30)	89 419	1.8
9	Chronic liver disease (K70, K73, K74)	72 379	1.5
10	Diseases of kidney and ureter (N00-N29)	66 131	1.3
…	All other causes (residual)	875 898	17.7
*(11*	*Intentional self-harm (suicide) (X60-X84, Y87.0)*	*57 715*	*1.2)*
**MALE**			
**…**	**All causes of death (A00-R99, V01-Y89)**	**2 461 153**	**100.0**
1	Diseases of the circulatory system (I00-I99)	838 133	34.1
2	Malignant neoplasms (C00-C97)	731 256	29.7
3	Chronic lower respiratory diseases (J40-J47)	95 043	3.9
4	Accidents (V01-X59, Y85, Y86)	90 382	3.7
5	Pneumonia (J12-J18)	58 392	2.4
6	Dementia (F01, F03)	51 552	2.1
7	Chronic liver disease (K70, K73, K74)	49 136	2.0
8	Diabetes mellitus (E10-E14)	49 097	2.0
*9*	*Intentional self-harm (suicide) (X60-X84, Y87.0)*	*44 461*	*1.8*
10	Diseases of kidney and ureter (N00-N29)	30 305	1.2
…	All other causes (residual)	421 966	17.2
**FEMALE**			
**...**	**All causes of death (A00-R99, V01-Y89)**	**2 483 898**	**100.0**
1	Diseases of the circulatory system (I00-I99)	994 552	40.0
2	Malignant neoplasms (C00-C97)	575 263	23.2
3	Dementia (F01, F03)	107 353	4.3
4	Chronic lower respiratory diseases (J40-J47)	68 899	2.8
5	Accidents (V01-X59, Y85, Y86)	61 954	2.5
6	Alzheimer disease (G30)	61 886	2.5
7	Pneumonia (J12-J18)	59 901	2.4
8	Diabetes mellitus (E10-E14)	59 553	2.4
9	Diseases of kidney and ureter (N00-N29)	35 825	1.4
10	Chronic liver disease (K70, K73, K74)	23 240	0.9
...	All other causes (residual)	435 472	17.5
*(13*	*Intentional self-harm (suicide) (X60-X84, Y87.0)*	*13 229*	0.5)

… – Category not applicable

**Table 2** and **Table 3** show the “top-10” leading causes of mortality in Europe (EU28) in 2014, for all age groups combined, selected from the two lists in **Table 1**. When using the list where diseases of the circulatory system, cancers and accidents were grouped, the first five leading causes were diseases of the circulatory system, which accounted for 37.1% of all deaths, cancers (26.4%), chronic lower respiratory diseases (3.3%), dementia (3.2%), and accidents and (3.1%). In the second list (**Table 3**), where we split the three categories for circulatory system diseases, cancers, and accidents, the five leading causes were cerebrovascular diseases (8.5%), malignant neoplasm of trachea, bronchus and lung (5.5%), acute myocardial infarction including subsequent myocardial infarction (4.7%), dementia (3.2%), and malignant neoplasm of colon, rectosigmoid junction, rectum, anus and anal canal (3.1%). In 2014, the 10 leading causes of deaths accounted for 82% of all deaths in EU-28 according to our first ranking list (**Table 2**), and corresponding for ranking list number two accounted for 35% (**Table 3**).

**Table 3:** The 10 leading causes of death selected from List II (see Table 1), by sex: 28 European Union member countries (EU-28), 2014

Rank	Split: Circulatory diseases; cancers; accidents (list II)	2014		
**Causes of death (ICD-10)**	**Deaths**	**%**	
**…**	**All causes of mortality (A00-R99, V01-Y89)**	**4 945 366**		**100.0**	
1	Cerebrovascular diseases (I60-I69)	421 558		8.5	
2	Malignant neoplasm of trachea, bronchus and lung (C33, C34)	271 816		5.5	
3	Acute myocardial infarction including subsequent myocardial infarction (I21, I22)	231 852		4.7	
4	Dementia (F01, F03)	158 906		3.2	
5	Malignant neoplasm of colon, rectosigmoid junction, rectum, anus and anal canal (C18-C21)	152 209		3.1	
6	Pneumonia (J12-J18)	118 295		2.4	
7	Diabetes mellitus (E10-E14)	108 654		2.2	
8	Malignant neoplasm of breast (C50)	93 490		1.9	
9	Alzheimer disease (G30)	89 419		1.8	
10	Malignant neoplasm of pancreas (C25)	83 325		1.7	
…	All other causes (residual)	3 215 842		65.0	
*(15*	*Intentional self-harm (suicide) (X60-X84, Y87.0)*	*57 715*		*1.2)*	
**MALE**					
**…**	**All causes of death (A00-R99, V01-Y89)**	**2 461 153**		**100.0**	
1	Malignant neoplasm of trachea, bronchus and lung (C33, C34)	184 816		7.5	
2	Cerebrovascular diseases (I60-I69)	175 193		7.1	
3	Acute myocardial infarction including subsequent myocardial infarction (I21, I22)	134 771		5.5	
4	Malignant neoplasm of colon, rectosigmoid junction, rectum, anus and anal canal (C18-C21)	83 841		3.4	
5	Malignant neoplasm of prostate (C61)	74 016		3.0	
6	Pneumonia (J12-J18)	58 392		2.4	
7	Dementia (F01, F03)	51 552		2.1	
8	Chronic liver disease (K70, K73, K74)	49 136		2.0	
9	Diabetes mellitus (E10-E14)	49 097		2.0	
10	*Intentional self-harm (suicide) (X60-X84, Y87.0)*	44 461		1.8	
…	All other causes (residual)	1 555 878		63.2	
**FEMALE**					
**...**	**All causes of death (A00-R99, V01-Y89)**	**2 483 898**		**100.0**	
1	Cerebrovascular diseases (I60-I69)	246 348		9.9	
2	Dementia (F01, F03)	107 353		4.3	
3	Acute myocardial infarction including subsequent myocardial infarction (I21, I22)	97 049		3.9	
4	Malignant neoplasm of breast (C50)	92 532		3.7	
5	Malignant neoplasm of trachea, bronchus and lung (C33, C34)	86 993		3.5	
6	Malignant neoplasm of colon, rectosigmoid junction, rectum, anus and anal canal (C18-C21)	68 362		2.8	
7	Alzheimer disease (G30)	61 886		2.5	
8	Pneumonia (J12-J18)	59 901		2.4	
9	Diabetes mellitus (E10-E14)	59 553		2.4	
10	Malignant neoplasm of pancreas (C25)	41 520		1.7	
...	All other causes (residual)	1 562 401		62.9	
*(23*	*Intentional self-harm (suicide) (X60-X84, Y87.0)*	13 229		0.5)	

… – Category not applicable

The two methods we used for ranking the European data affected suicide. Suicide was the 11^th^ and 15^th^ leading cause in List I (**Table 2**) and List II (**Table 3**), respectively. When looking at the data by sex, suicide for males was ranked at the ninth and the tenth leading cause of death in the two ranking lists. For females, suicide was number 13 in the first and 23 in the second list, respectively.

**Figure 1:**
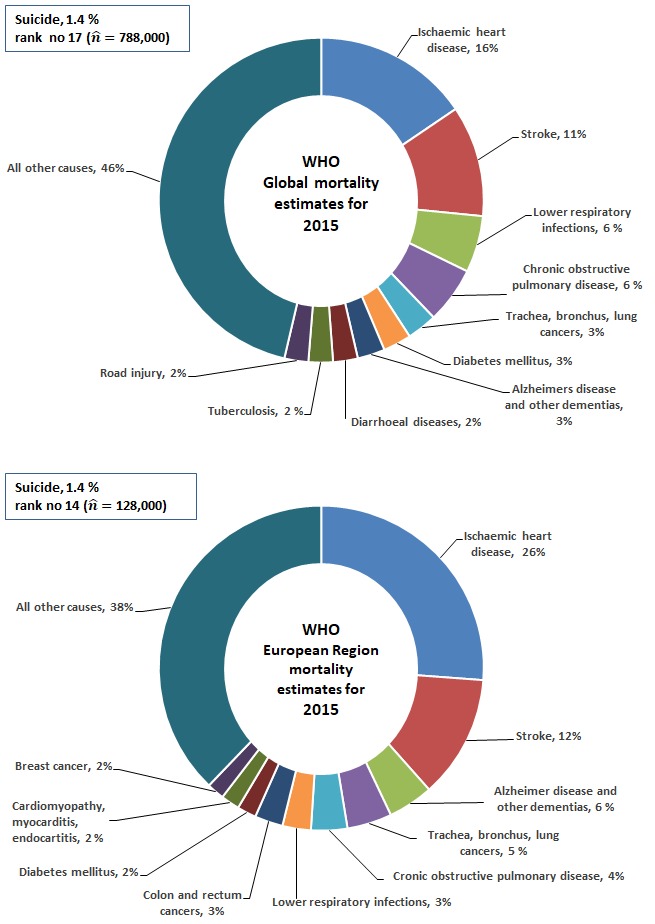
Ten leading causes of mortality globally (upper panel) and in Europe (lower panel).

The global mortality estimates from the World Health Organization for 2015 indicates that two of the ten leading causes of death were subcategories of circulatory diseases, and one cancer disease (trachea, bronchus and lung) (**Figure 1**). Alzheimer`s disease and other dementias was the seventh leading cause of mortality, and road injury was ranked as the tenth leading cause, after diarrhoeal diseases, and tuberculosis. Road injury includes all land transport accidents (traffic and non-traffic) except for pedestrian injured in collision with railway train, and occupants on railway train, streetcar and special industrial and agricultural vehicle injured in transport accidents [20,21]. In 2015, the 10 leading causes of mortality globally, accounted for 54% of all estimated deaths, and corresponding figure for the WHO European region was 62% (**Figure 1**).

In the WHO European Region, three of the ten leading causes of mortality were circulatory system diseases (ischemic heart disease, and stroke, and cardiomyopathy, myocarditis, endocarditis), and three were cancers (trachea, bronchus and lung cancers, and colon and rectum cancers, and breast cancer).

Globally, suicide was ranked as the 17^th^ leading cause of mortality in 2015 by WHO [19], and in the WHO European Region, suicide was ranked as the 14^th^ leading cause (**Figure 1**). According to the WHO mortality estimated for 2000-2015 [19], the ranking of suicide changed significantly in the European Region from being the seventh leading cause in 2000 to be the 14^th^ in 2015 (the estimated rates for the two years changed from 19.9 to 14.1 per 100 000 population). According to the latest GHE 2015, globally, suicide was ranked as the 15^th^ leading cause in 2000 (17^th^ in 2015), and the estimated suicide rates decreased from 12.2 per 100 000 in 2000 to 10.7 in 2015 [19]. This corresponds to 748 000 estimated number of suicide deaths in 2000, and 788 000 in 2015 [19]. The GHE 2015 (estimates for 2015) [19], and the previous GHE 2014 (estimates for 2012) [22], presents significant different information regarding the estimated number and rates of suicide for the year 2000. According to GHE 2014, the number of suicide deaths was 883 000 in 2000, and 804 000 in 2012 (the estimates rates were 14.4 per 100 000 and 11.4, respectively), and suicide was ranked as the 14^th^ and 15^th^ leading cause in 2000 and 2012, respectively [22]. Globally, both GHE 2015 [19], and GHE 2014 [22] indicate decreasing suicide rates over time.

Regarding the quality of the 2014 data for the 28 European countries, 3.5% of all deaths (about 174 000 deaths), were allocated to symptoms, signs, ill-defined conditions and unknown cause (excluding the subcategory sudden infant death syndrome).

## DISCUSSION

### Main findings in context

We have shown how two different methods gave different leading underlying causes of mortality in Europe, and how this affected the rank order of suicide. Based on the Eurostat’s short list of causes of death, we selected rankable causes and used different levels of details for three cause categories: circulatory system diseases, malignant neoplasms, and accidents. These three causes ranked high when using aggregated groupings, all among the top five leading causes (**Table 2**). The importance of circulatory diseases (accounted for 38% of all deaths in EU-28 in 2014), and cancers (26% of all deaths), was shown also when these disease groups were split into more specific diagnoses (6 out of the 10 leading causes were subgroups of circulatory diseases, and cancers, **Table 3**). When we disaggregated unintentional injury deaths, no subcategory of accident mechanism (eg, transport, falls and poisoning) was among the 10 leading causes.

These results were in accordance with previous studies from Europe: Griffiths et al. [12], demonstrated how the leading causes in lists of the top ten in England and Wales, in 2003, varied as a consequence of using grouped or more detailed categories of malignant neoplasms and accidents. In the England and Wales study, the authors grouped suicide together with the majority of injury and poisoning deaths of undetermined intent because most deaths coded to undetermined intent in England and Wales are thought to be deaths from self-harm for which the coroner returns an open verdict (insufficient information that deceased intended to die) [12]. Despite this broader grouping, suicide was not among the top ten leading causes in England and Wales, for males or females. Another study from Becker et al [7], showed how the list with leading causes of death changed when using the broad category for all cancers, and when cancers were disaggregated by site, based on data for western Europe, 2001. Suicide ranked as the 14^th^ leading causes of death in the western part of Europe in 2001, and accounted that year for 1.7% of all deaths (for males and females suicide was ranked as the 9^th^ and 26^th^, respectively) [7].

In the United States, in 2015 (the most recent year of available death data), suicide was ranked as the 10^th^ leading cause of death [23,24], and this rank has been unchanged since 2008 [25]. In 2015 suicide accounted for 1.6% of all deaths in the United States (number of suicide deaths was 44 193, and the crude rate was 13.7/100 000 population) [24,26]. In the last 40 years, since 1975, suicide has been among the top 12 leading causes of death in the US [24].

### Suicide globally

Regarding suicide globally, the WHO publication on global suicide prevention [8], stated suicide was the 15^th^ leading cause of mortality globally in 2012. GHE 2014 [22] was used as data source in this WHO report, and necessarily corresponding to what we have reported from the same source. However, the uncertainty of the global cause of death estimates is great. One example: According to estimates in GHE 2015 [19] the magnitude of suicide deaths globally in 2000 was over-estimated by 135 000 deaths in GHE 2014 [22]; 748 000 and 883 000 suicide deaths, respectively. This relatively large difference in the estimated number of suicides in 2000 only had a minor effect of the rank (suicide moved down from 14^th^ to 15^th^ rank in GHE 2014 and GHE 2015 in the year 2000, respectively). A newer report on mental health globally states that suicide rates are increasing globally [27]. However, and as we presented, this is not in line with the two most recent estimates of the GHE [19,22], which both indicate that global suicide rates have decreased. We may add that Bertolote and De Leo [28] have also shown decreasing trends in suicide globally, when assessing trends from 1992 to 2009 using available mortality data in WHO’s data bank from 62 countries. WHO has previously used a similar method to shown that suicide rates were quite stable during a period from 1970 to 1996, for the 39 countries for which complete data was available [29].

Leading causes of mortality (and morbidity) can give useful information supporting the advance of public health, but are only a starting point for supplemental analysis [7] eg, on cause specific mortality rates, trends over time, and causes by age and sex. Without a clear understanding of what the ranking means and its inherent limitations [7,9,10], it is easy to cross the line between good use and misuse of statistics [30,31]. There are many ways to misuse statistics in science, and one of the most common forms of the misrepresentation of data are to exaggerate the significance of results [30]. By saying that a specific cause is “among the leading causes of mortality”, without presenting the numbers and the percentage of all deaths, this may result in misinterpretation and undermine the public`s trust in public health science.

### Limitations

This study has limitations. First, the rankings do not necessarily present the causes of mortality of greatest public health importance. Some causes of public health importance may not be among the ranked causes because they either are incorporated into broader categories (eg, road traffic accidents), or they appears infrequently in official mortality statistics. This is the case for conditions that to some extent should be used as the underlying cause, when more than one condition is reported on the death certificate (eg, alcohol conditions, diabetes, and mental disorders). It is a common misunderstanding, eg, in medical research, that mortality statistics mirror the prevalence of specific conditions reported on the death certificate, and judge cases as “misclassified” if official mortality statistics do not match the number of cases identified on the death certificate [32]. This means that many studies assessing quality of mortality statistics are flawed [10,32]. We must always keep in mind that the rankings present underlying causes of death [15], among those cause categories available to be ranked [7,9], which implies that only one cause is used when two or more diseases, injuries or other conditions are recorded on the death certificate. Further, quality of mortality statistics may affect the ranking (and all other mortality analysis). The debate regarding quality [17], is often a mixed debate regarding completeness, and misclassification, and also the terms used in ICD. For example, Rockett and co-authors [33] questioned the dichotomy between suicide and unintentional (accidents) deaths, and argued the need for statistics that better reflect the burden of deaths due to deliberate, self-destructive behavior (self-injury deaths). This category group all suicides together with a major proportion of accidental - and undetermined drug intoxication deaths [33]. The self-injury category would constitute the eighth leading cause of death in US [33], and the subject is part of an ongoing debate regarding classification of suicide and drug intoxication deaths in US [34]. In addition, the number of deaths allocated to symptoms, ill-defined and unknown causes of death is of special concern for the quality of mortality data [12,17]. When it comes to injury deaths, lack of information on the external cause of injuries and poisonings may overestimate number of accidents and underestimate suicide [12,17,35,36]. Third, a change in the ranking of a specific cause over time may occur even though the proportion of deaths classified to that cause did not change [9]. Similarly, two populations with different risk levels (rates) for a specific cause (eg, suicide), may have the same rank for this condition [9]. Introduction of new revisions of the classification system and coding rules is another factor that can substantially affect the ranking. Moving to ICD-10 in 1999 in US affected the ranking of five out of the ten leading causes: diabetes, influenza and pneumonia, Alzheimer`s disease, suicide, and chronic liver disease and cirrhosis [37].

Finally, we did not assess leading causes in different age groups. In general, the pattern differs in the way that injury deaths (accidents, suicide, homicide) account for relatively more deaths in younger age groups, in contrast to older age groups [9,12].

### CONCLUSIONS

To ensure that ranking causes of death is not driven by political motives and funding considerations, standard methods and official tabulation lists should be used. A ranking of causes of death should always present the proportion of all deaths and number of deaths allocated to the ranked causes, similarly for the residuals not covered, and a data quality indicator.
